# Effects of a Lactobacilli Probiotic on Reducing Duration of URTI and Fever, and Use of URTI-Associated Medicine: A Re-Analysis of a Randomized, Placebo-Controlled Study

**DOI:** 10.3390/microorganisms9030528

**Published:** 2021-03-04

**Authors:** Tatiana Altadill, Jordi Espadaler-Mazo, Min-Tze Liong

**Affiliations:** 1 AB-Biotics SA, Sant Cugat del Vallès, 08172 Barcelona, Spain; rd@ab-biotics.com; 2 Department of Bioprocess Technology, School of Industrial Technology, University Sains Malaysia, Penang 11800, Malaysia

**Keywords:** probiotic, URTI, fever, *Lactobacillus plantarum*, immunity

## Abstract

We previously reported on the effects of *Lactoplantibacillus plantarum* DR7 on reducing Upper Respiratory Tract Infections (URTI) symptoms’ score and frequency in 109 adults upon a 12-week consumption at 10^9^ colony-forming units (CFU)/day, but several limitations were detected in the publication. Thus, the present study re-analyzed some data with the aim to address some of these weaknesses, and presents new data on duration of URTI and consumption of URTI-associated medication, as compared to the placebo. Our re-analyses found probiotic administration significantly reduced the proportion of patient days of URTI and of fever (all *p* < 0.05). Recent history of URTI was a prevalent co-factor in affecting duration of URTI symptoms and fever, while other demographic and clinical factors had no influence. Exploratory analyses suggested probiotic had an earlier benefit in patients without a recent history of URTI compared to those with a recent history of URTI. Therefore, recent history of infections could have a modulatory effect on probiotic efficacy. Average number of months with reported use of URTI-related medication was 3.4-times lower in the probiotic group as compared to placebo (*p* = 0.016) during the intervention. Taken together, our present new data further support previous findings that DR7 probiotic had a beneficial effect on URTI.

## 1. Introduction

Upper respiratory tract infection (URTI) is likely the most frequent illness in adults [1]. While the primary symptoms of URTI include nasal stuffiness and discharge, sneezing, sore throat, and cough, the onset of fever is common, as accompanied by some cases of clinical manifestations which may vary by age. Paranasal sinusitis is the most frequent complication of URTI in this population [2]. Adults have two to four episodes per year [3], leading to a rise in absentia at work, causing much economical and financial burden on the patients and families, workplace organizations, healthcare, and insurance providers. 

URTI is most commonly caused by viruses, namely rhinoviruses, coronaviruses, adenoviruses, respiratory syncytial virus, influenza, and parainfluenza virus [4], indicating that the host’s immune system and responses play crucial roles in both preventing URTI and shortening the recovery period upon an infection [5]. Various dietary interventions to modulate the host’s immune systems have been proposed, ranging from supplementations with specific nutrients such as vitamin C or D [6,7] to global dietary advice aimed at increasing intake of vitamins, minerals, and antioxidants compounds, to increasing consumption of fiber and vegetables [8]. Another dietary strategy involves the use of probiotics, which are beneficial microorganisms that exert health benefits to the hosts upon consumption in adequate amounts [9]. Meta-analyses indicate probiotics can be useful for prevention of URTIs, but a significant degree of heterogeneity between studies and strains is noted [10]. This is to be expected, since probiotic immunological effects are thought to be strain-specific [9]. As probiotics are consumed orally to reach the gastrointestinal tract, these microorganisms are postulated to benefit the hosts via affecting gut microbiota of the hosts and/or signaling to the immune cells in the gut. Probiotics have been reported to alter the profiles of gut microbiota via sharing genes and metabolites; and supporting the growth and proliferation of certain groups within the microbiota, where all these ultimately influence the immunity landscapes of gut epithelial cells [11]. 

We have previously reported that one strain of probiotic, *Lactoplantibacillus plantarum* DR7 (formerly known as *Lactobacillus plantarum* DR7), exerted immune modulatory effects, primarily via activation of natural killer (NK) cells leading to reduced duration of nasal symptoms and frequency of URTI incidences in 109 adults upon a 12-week consumption at 10^9^ CFU/day [12]. This same strain was also shown to modulate gut microbiota in human subjects [13]. The initial publication from Chong and coworkers had several shortcomings; (i) emphasis of a symptom-based score, where a higher score could indicate both longer duration of symptoms and higher frequency of symptoms, thus unable to distinguish one from the other; (ii) grouping of symptoms into three main categories namely nasal, pharyngeal, and flu-like, thus did not include a global score; (iii) only episodes of URTI were presented, without quantifying the actual effect on episode duration; and (iv) effect of baseline demographic and clinical characteristics was not assessed.

Thus, the present study aimed to address some of these weaknesses, via a re-analysis of data from the previously described study population [12] to better assess and present the effect of *L. plantarum* DR7 on the duration of URTI, as measured by patient days of URTI and patient days of fever, as compared to the placebo. Moreover, the study also aimed at assessing the impact of *L. plantarum* DR7 on URTI-related medication use.

## 2. Materials and Methods

### 2.1. Study Design and Population

The study design population has been described elsewhere in detail [12]. Briefly, this was a randomized, double-blinded, placebo-controlled study, enrolling healthy adult men and women. Subjects (*n* = 124) were allocated 1:1 to receiving probiotic one sachet per day (containing 10^9^ CFU of *L. plantarum* DR7 in a maltodextrin carrier, while cell count was determined using the serial dilution pour plate method on de Mann, Rogosa, Sharpe (MRS) agar at 37 °C and incubated anaerobically) or an indistinguishable placebo sachet per day (containing maltodextrin carrier only) for 12 weeks. The study design, patients flow through the study (CONSORT flowchart), randomization process approval by the human research ethical committee of Universiti Sains Malaysia, and consent attainment process were as previously reported [12]. The study adhered to the tenets of the Declaration of Helsinki.

### 2.2. Study Outcomes

Monthly information on URTIs was collected at baseline and after 4, 8, and 12 weeks with a questionnaire validated in English, Malay, and Chinese [14]. The questionnaire recorded days of nasal (e.g., sneezing, runny nose), pharyngeal (e.g., sore throat, cough), and general flu symptoms (e.g., fever, body aches, headache), as well as total number of URTI episodes occurring in the 4 weeks before the assessment. Data on use of medication for URTIs (antipyretics, antibiotics, antihistamines, antitussives) in the previous 4 weeks was also collected at baseline and after 4, 8, and 12 weeks, which was recorded as yes/no.

The main study outcome was the proportion of patient days of URTIs. Based on the aforementioned questionnaire, the duration of URTIs was defined in two ways: (i) The duration of the longest-lasting symptom reported; and (ii) the average duration of all symptoms reported. The secondary outcomes were the proportion of patient days with fever (≥37 °C) and number of months in which URTI-related medication was used.

### 2.3. Statistical Analysis

The analysis population consisted of the 109 subjects providing monthly questionnaires. The SPSS^®^ Statistics^®^ v. 20.0 (IBM^®^, Armonk, NY, USA) statistics program was used for statistical analyses. Data normality was checked with Shapiro–Wilk test. Pearson’s chi-squared test was used to compare categorical data, Student T-test was used for continuous parametric data, and Mann–Whitney test was used for continuous non-parametric data. Because an imbalance was observed in baseline data regarding recent history of URTIs within the previous month, analyses of study outcomes (patient days of URTI and patient days of fever) were performed both unadjusted and adjusted for this baseline variable, as recommended by the European Medicines Agency (EMA) guideline ([EMA/CHMP/295050/2013]). More precisely, unadjusted analyses were performed with Pearson’s chi-squared test (as above indicated for categorical data), while adjusted analyses were performed using the Mantel–Hanszel test. Spearman test was used to assess pairwise correlation between study outcomes and baseline clinical and demographic parameters (age, sex, body mass index, urban vs. rural living, blood hemoglobin, white cell count, and URTI episodes in the 4 weeks prior to study enrolment). Correlation to smoker status was not assessed because only one individual in the recruited population reported being a regular smoker. In exploratory analyses, Bonferroni multiplicity correction was applied within each study outcome among sub-populations (i.e., subjects with and without prior URTI) and/or different times (month 1: weeks 0 to 4; month 2: weeks 4 to 8; and month 3: weeks 8 to 12), or in correlation analyses against baseline clinical and demographic parameters (these *p*-values are denoted as p_corr_). All statistical tests were two-sided.

## 3. Results

### 3.1. Study Population

The analysis population consisted of the 109 subjects providing monthly questionnaires for 3 months, 56 in the probiotic group and 53 in the placebo one (Table 1). This amounts to 9156 patient days available for analysis in the study population, 4452 in the placebo group and 4704 in the probiotic group. Median age was 29 years old (range 21 to 63) and 71% were women, with insignificant difference between groups. There was only one smoker, and few (6%) had been hospitalized or reported having used URTI-related medication (8%) within the previous month. Overall, there were insignificant differences between groups regarding these variables. Conversely, 35% reported having one or more URTI episodes within the previous month, with a statistical trend towards more episodes in the probiotic group than in the placebo one (Mann–Whitney *p* = 0.059). Therefore, subsequent analyses were performed both unadjusted and adjusted for the number of URTI episodes within the previous month.

### 3.2. Effect of Clinical and Demographic Characteristics at Baseline on Study Outcomes

A first part of the analysis included the study of the correlation between baseline characteristics and the study outcomes. Of all clinical and demographic baseline parameters analyzed (age, sex, body mass index, urban vs. rural living, blood hemoglobin, white cell count, and URTI episodes in the 4 weeks prior to study enrolment), only history of recent URTI significantly correlated to study outcomes, the effect being robust to multiplicity correction: days of URTI according to longest-lasting symptom (rho = 0.29, p_corr_ = 0.014), days of URTI according to average duration of symptoms (rho = 0.28, p_corr_ = 0.028), and days of fever (rho = 0.28, p_corr_ = 0.021). A statistical trend (rho = 0.24, p_corr_ = 0.077) was found between recent hospitalization and days of URTI according to average duration of symptoms, but not days of URTI according to longest-lasting symptom or days of fever. Conversely, no significant correlations were found between baseline parameters and use of URTI-related medication during the 12-week intervention, including history of recent URTI (p_corr_ > 0.10 for all). See Table A1 in Appendix A.

### 3.3. Effect of Probiotic Treatment on Patient Days of URTI and of Fever

During the 12-week intervention period, the proportion of patient days of URTI reported in the probiotic group was significantly lower than in the placebo one, both when considering the duration of URTI as the longest-lasting symptom or as the average duration of URTI symptoms (*p* < 0.05, Table 2). The same effect was observed when considering the total number of days with fever (*p* < 0.05). Moreover, significances were markedly increased upon adjusting based on baseline variables of prior URTI history before inclusion in the study (*p* < 0.001 in all three outcomes). Therefore, the effects of the probiotic in reducing patient days of URTI and of fever are robust.

### 3.4. Differential Effect Based on Patient History of Recent URTIs and Time

Given the effect of history of recent URTI on some study outcomes, the study population was subdivided according to this baseline variable in exploratory analysis to study the effect of time during follow-up (Table 3). Among those without recent history of URTI, subjects in probiotic group experienced a lower proportion of patient days of URTI during the first two months compared to placebo, as counted both using the longest-lasting symptom and the average duration of symptoms (p_corr_ ≤ 0.005 in all cases). The effect on patient days of URTI faded away during the last month because the proportion of patient days in the placebo group kept going down during the study and reached similar levels to probiotic on the third month of intervention. Similarly, the effect on patient days of fever was very significant on the first month (p_corr_ < 0.001) then faded away because of incidence of fever dwindling in placebo group, although a statistical trend favoring DR7 probiotic was still detected on the third month, even after accounting for multiplicity correction (p_corr_ = 0.092). 

While patients with recent history of URTI displayed a nominally higher proportion of patient days of URTI at baseline in the probiotic group than placebo group, this trend was reversed along the intervention period and attained significance in the last month of intervention for patient days of URTI defined as the average duration of symptoms (p_corr_ = 0.01) but not when defined as per the longest-lasting symptom, after accounting for multiplicity correction. Patient days of fever were also significantly lower in probiotic group on the third month (*p* = 0.006), accounting for multiplicity correction.

After splitting the population by history of recent URTI, no significant differences were found regarding baseline demographic and clinical characteristics (age, sex, body mass index, urban vs. non-urban living, blood hemoglobin and white cell count, and recent hospitalization history) when comparing probiotic and placebo groups (See Table A2 in Appendix B). 

While the effects of probiotic intervention in reducing patient days of URTI and fever was faster in the population without recent history of URTI (first and second month of intervention), such an effect was also evident albeit later (third month of intervention) in the population with recent history of URTI. It must be noted that the robustness of this observation remained, amid multiplicity correction.

### 3.5. Effect on URTI-Related Medication

Overall, there was a significant 3.4-fold reduction in average number of months with reported use of URTI-related medication in probiotic group vs. placebo (0.14 ± 0.05 vs. 0.49 ± 0.12; *p* = 0.016) during the 12-week intervention. In exploratory analysis by month, a statistical trend could still be detected favoring DR7 probiotic against placebo on the second and third months of intervention, even after accounting for multiplicity correction (p_corr_ = 0.069 and 0.062, respectively).

## 4. Discussion

Previous studies utilizing dietary interventions such as that with probiotics, have reported beneficial effects against URTI [15,16]. Considering that URTI is the most common cause of absentia at work for adults, the current re-analysis of data emphasized on the duration factor, for both duration of URTI and fever which were not presented in the previous study [12]. Among all URTI symptoms, fever has been reported to be both the most objective symptom and the most frequent drive for school and work absentia [17].

The current re-analysis disregarded the type of symptoms, which were categorized into three in the previous study (pharyngeal, nasal, or flu-like), and focused instead on the aspects of duration. Considering that the exact duration of the URTI is often difficult to be precisely established, two alternative definitions are used to assess the robustness of the findings, including (i) duration of longest symptom and (ii) average duration of symptoms. Our present data showed that similar effects are observed using both definitions of URTI with the same observation for fever, indicating that the findings of the reanalysis are robust.

Our present data showed that *L. plantarum* DR7 reduced the proportion of patient days of URTI as compared to the placebo for all parameters studied i.e., duration of URTI as the longest-lasting symptom, average duration of URTI symptoms, and total number of days with fever. Meanwhile, exploratory analyses suggested the efficacy of *L. plantarum* DR7 at different time-points during the 12-week intervention period was affected by the recent history of URTI. Accounting for multiplicity of analyses, *L. plantarum* DR7 still had a significant effect on patient days of URTI and of fever during the first two months of the intervention in patients without recent history of URTI. Conversely, *L. plantarum* DR7 managed to achieve a significant effect on the third month of intervention as compared to the placebo in patients with recent history of URTI, despite accounting for multiplicity of analyses. Taken altogether, these data showed that although *L. plantarum* DR7 exerted beneficial effects, the actions for such an effect to take place may be dependent on two crucial factors i.e., the patients’ recent history of infections and the start-point of administration of *L. plantarum* DR7. Should *L. plantarum* DR7 be administered prior to an infection, the severity of URTI may be reduced faster, as compared to the administration of *L. plantarum* DR7 amid an active infection that has already occurred. 

Our present data also showed that the administration of DR7 reduced the use of URTI-related medication as compared to the placebo counterpart during the second and third months of intervention, where this effect was also observed cumulatively upon consideration of the entire 12-week period. While a segregation on types of URTI-related medication could not be performed because of unavailability of such data, antibiotics are commonly prescribed for URTI in additional to antipyretics and antihistamines. The use of antibiotics has been a major health concern attributed to increased risks of antibiotic resistance among gut microbiota of the hosts, where WHO has listed antibiotic resistance as one of the biggest threats to global health and food security [18]. Here, we present another perspective of probiotics, involving the reduced need for prolonged administration of antibiotics, which is attributed to a faster recovery rate as compared to placebo, in hope to serve as a natural strategy against increased risks of antibiotic resistance. The observed difference in time to efficacy depending on recent history of URTI did not correlate to demographic and clinical factors such as age, sex, body mass index, urban vs. rural living, blood hemoglobin and white cell count, and recent hospitalization. Therefore, it seems the recent history of URTI per se could be the causative factor. In this regard, one of the putative mechanisms of action of *L. plantarum* DR7 involves the activation of NK-cells [12], and chronic infection in general and frequent URTIs in particular have been shown to lead to NK cell exhaustion [19,20]. 

On the other hand, probiotics may exert immunomodulatory effects in viral and bacterial infections through modulation of cytokine levels [21,22]. Particularly, DR7 reduced pro-inflammatory cytokine IFN-γ and increased anti-inflammatory cytokines IL-10 in plasma, and targeted the tryptophan-5HTT-kynurenine pathway [12] via downregulation of IDO and TDO [13]. It can be hypothesized that patients with recent history of URTI may have an overactivation of IDO and a higher basal concentration of local and systemic cytokine levels, thus needing a longer period of DR7 intake to normalize cytokines and the tryptophan-5HTT-kynurenine pathway.

Finally, respiratory infections have been reported to result in dysbiosis in the gastrointestinal tract [23]. We have previously reported the ability of *L. plantarum* DR7 in modulating gut microbiota profiles in adults [13], but this probiotic could need more time to modulate gut microbiota profiles of patients with recent history of URTI, prior to activation of hosts’ immune system, leading to a slower time to exert benefits against any observable URTI symptoms.

This study also has some limitations worth mentioning. First, physical exercise is known to influence immune status [24], but data on the regular practice of high intensity exercise was not available from the original study, and therefore its impact on the effect of the probiotic could not be assessed. Second, the diagnosis of URTIs was based on a questionnaire instead of a direct examination by a specialist, thus lowering the accuracy of the diagnosis because of patients’ subjectivity. However, the results based on patient days of URTI show good agreement to those based on patient days of fever, which is a more objective parameter. Moreover, in dealing with URTIs, adults often do not seek for a medical visit, rather relying on over-the-counter medication. Thus, the procedure followed in this study approaches this real-life practice. Finally, the study did not include elderly subjects, which are an interesting target for URTI prevention among adults.

## 5. Conclusions

Administration of probiotic strain *L. plantarum* DR7 significantly reduced patient days of URTI and of fever. The results are robust to the baseline imbalance in history of recent URTI between groups. The administration of DR7 also reduced the use of URTI-related medication which may serve importance against antibiotics resistance. Additional exploratory analyses suggested the efficacy of a probiotic, depending on its core mechanism of actions, may be dependent on the patients’ recent history of infection and the start-point of administration of probiotic. 

## Figures and Tables

**Table 1:** Baseline characteristics of the 109 adult subjects randomly assigned to 12 weeks of double-blind treatment with either 10^9^ CFU/day *L. plantarum* DR7 (*n* = 56) or placebo (*n* = 53).

Parameters	Probiotic (*n* = 56)	Placebo (*n* = 53)	*p*-Value
Age (year, median and range)	30.5 (21–58)	28.0 (21–63)	0.767
Sex (num. female, %)	39 (69.6%)	38 (71.7%)	0.814
Body Mass Index (median and range)	22.4 (17.7–35.4)	22.2 (16.7–39.1)	0.402
Smoker (num. yes, %)	1 (1.8%)	0 (0%)	0.331
Hospitalization within 4 weeks (num. yes, %)	3 (5.4%)	4 (7.5%)	0.643
Blood hemoglobin (g/L, mean and SD)	137.2 (15.9)	135.8 (15.6)	0.624
White cell count (×10^9^/L, mean and SD)	7.2 (1.8)	7.3 (2.4)	0.757
Urban living (num. yes, %)	29 (51.8%)	27 (50.9%)	0.930
URTIs within 4 weeks			
>2 episodes	3 (5.4%)	1 (1.9%)	0.059
2 episodes	5 (8.9%)	2 (3.8%)	
1 episode	16 (28.6%)	11 (20.8%)	
None	32 (57.1%)	39 (73.6%)	
URTI-related medication within 4 weeks (num. yes, %)	5 (8.9%)	4 (7.5%)	0.794

**Table 2:** Proportion of patient days for longest lasting symptom, average duration and fever from 109 adult subjects randomly assigned to 12 weeks of double-blind treatment with either 10^9^ CFU/day *L. plantarum* DR7 (*n* = 56) or placebo (*n* = 53).

Parameter	Probiotic	Placebo	Unadjusted *p*-Value ^A^	RR (95% CI)	Adjusted *p*-Value ^B^
Patient days of URTI (longest lasting symptom)	278 of 4704 (5.9%)	316 of 4452 (7.1%)	0.021	0.83 (0.71–0.97)	<0.001
Patient days of URTI (average duration of symptom)	183 of 4704 (3.9%)	213 of 4452 (4.8%)	0.036	0.81 (0.67–0.99)	<0.001
Patient days of fever	88 of 4704 (1.9%)	118 of 4452 (2.7%)	0.012	0.71 (0.54–0.93)	<0.001

*p*-values indicate differences between treatment groups based on ^A^ Pearson’s Chi-square test; ^B^ Mantel–Haenszel test.

**Table 3:** Proportion of patient days for longest lasting symptom, average symptom duration and fever from 109 adult subjects randomly assigned to 12 weeks of double-blind treatment with either 10^9^ CFU/day *L. plantarum* DR7 (*n* = 56) or placebo (*n* = 53).

Parameter	Recent URTI	Group	Previous Month	Month 1	Month 2	Month 3
Patient days of URTI (longest lasting symptom)	No	Placebo (*n* = 39)	N/A	82 of 1092 (7.5%)	74 of 1092 (6.8%)	36 of 1092 (3.3%)
		Probiotic (*n* = 32)	N/A	31 of 896 (3.5%)	12 of 896 (1.3%)	33 of 896 (3.7%)
		*p*-value	-	<0.001 *	<0.001 *	1.000
	Yes	Placebo (*n* = 14)	76 of 392 (19.4%)	34 of 392 (8.7%)	41 of 392 (10.5%)	56 of 392 (12.5%)
		Probiotic (*n* = 24)	165 of 672 (24.6%)	89 of 672 (13.2%)	57 of 672 (8.5%)	56 of 672 (8.3%)
		*p*-value	0.366	0.172	1.000	0.196
Patient days of URTI (average duration of symptoms)	No	Placebo (*n* = 39)	N/A	58 of 1092 (5.3%)	52 of 1092 (4.8%)	22 of 1092 (1.9%)
		Probiotic (*n* = 32)	N/A	21 of 896 (2.3%)	9 of 896 (1.0%)	26 of 896 (3.0%)
		*p*-value	-	0.004 *	<0.001 *	1.000
	Yes	Placebo (*n* = 14)	52 of 392 (13.6%)	18 of 392 (4.6%)	26 of 392 (6.5%)	38 of 392 (9.7%)
		Probiotic (*n* = 24)	113 of 672 (16.8%)	56 of 672 (8.3%)	40 of 672 (5.9%)	32 of 672 (4.7%)
		*p*-value	0.884	0.158	1.000	0.010 *
Patient days of fever	No	Placebo (*n* = 39)	N/A	37 of 1092 (3.4%)	15 of 1092 (1.4%)	13 of 1092 (1.2%)
		Probiotic (*n* = 32)	N/A	2 of 896 (0.2%)	4 of 896 (0.4%)	2 of 896 (0.2%)
		*p*-value	-	<0.001 *	0.242	0.092 ^#^
	Yes	Placebo (*n* = 14)	37 of 392 (9.4%)	12 of 392 (3.1%)	11 of 392 (2.8%)	30 of 392 (7.7%)
		Probiotic (*n* = 24)	65 of 672 (9.7%)	38 of 672 (5.7%)	21 of 672 (3.1%)	21 of 672 (3.1%)
		*p*-value	1.000	0.377	1.000	0.006 *

* Significant after Bonferroni correction for multiplicity of comparisons; ^#^ Statistical trend after Bonferroni correction for multiplicity.

## Data Availability

The data presented in this study are available upon reasonable request from the corresponding author.

## Appendix A

**Table A1:** Significant correlations (Spearman test) between baseline characteristics and study outcomes.

Baseline Parameters	Patient Days of URTI (Longest Lasting Symptom)	Patient Days of URTI (Average Duration of Symptom)	Patient Days of Fever	Use of URTI-Related Medication
Age	p_corr_ > 0.10	p_corr_ > 0.10	p_corr_ > 0.10	p_corr_ > 0.10
Sex	p_corr_ > 0.10	p_corr_ > 0.10	p_corr_ > 0.10	p_corr_ > 0.10
Body Mass Index	p_corr_ > 0.10	p_corr_ > 0.10	p_corr_ > 0.10	p_corr_ > 0.10
Smoker	p_corr_ > 0.10	p_corr_ > 0.10	p_corr_ > 0.10	p_corr_ > 0.10
Hospitalization within 4 week	p_corr_ > 0.10	p_corr_ = 0.077 (rho = 0.24)	p_corr_ > 0.10	p_corr_ > 0.10
Blood hemoglobin	p_corr_ > 0.10	p_corr_ > 0.10	p_corr_ > 0.10	p_corr_ > 0.10
White cell count	p_corr_ > 0.10	p_corr_ > 0.10	p_corr_ > 0.10	p_corr_ > 0.10
Urban vs. rural living	p_corr_ > 0.10	p_corr_ > 0.10	p_corr_ > 0.10	p_corr_ > 0.10
History of recent URTI	p_corr_ = 0.014 (rho = 0.29)	p_corr_ = 0.028 (rho = 0.28)	p_corr_ = 0.021 (rho = 0.28)	p_corr_ > 0.10
URTI-related medication within 4 week	p_corr_ > 0.10	p_corr_ > 0.10	p_corr_ > 0.10	p_corr_ > 0.10

## Appendix B

**Table A2:** Baseline characteristics of the study population (Probiotic *n* = 56, Placebo *n* = 53), split by history of recent URTI (presence or absence of URTI within past 4 weeks). No significant differences were noted between interventions within each subpopulation.

Parameters	No Recent URTIProbiotic (*n* = 32)	No Recent URTI Placebo (*n* = 39)	*p*-Value	Recent URTI Probiotic (*n* = 24)	Recent URTI Placebo (*n* = 14)	*p*-Value
Age (y, median and range)	29.0 (21–58)	28.0 (21–63)	0.959	32.0 (21–48)	28.5 (22–52)	0.555
Sex (num. female, %)	21 (65.5%)	26 (66.7%)	0.927	18 (75.0%)	12 (85.7%)	0.441
Body Mass Index (median and range)	22.4 (18.4–31.3)	22.8 (16.8–39.1)	0.383	22.7 (17.7–35.4)	21.5 (16.7–35.2)	0.820
Smoker (num. yes, %)	0 (0%)	0 (0%)	1.000	1 (4.2%)	0 (0%)	0.445
Hospitalization within 4 week (num. yes, %)	1 (3.1%)	3 (7.7%)	0.410	2 (8.3%)	1 (7.1%)	0.897
Blood hemoglobin (g/L, mean and SD)	138.3 (15.2)	138.2 (16.1)	0.996	135.8 (17.1)	129.2 (11.4)	0.206
White cell count (×10^9^/L, mean and SD)	6.9 (1.8)	7.3 (2.0)	0.402	7.5 (1.8)	7.4 (3.3)	0.945
Urban living (num. yes, %)	15 (48.7%)	19 (46.9%)	0.878	14 (60.9%)	8 (57.1%)	0.825
URTIs within 4 week						
>2 episodes	0 (0%)	0 (0%)	1.000	3 (12.5%)	1 (7.1%)	0.444
2 episodes	0 (0%)	0 (0%)		5 (20.8%)	2 (14.3%)	
1 episode	0 (0%)	0 (0%)		16 (66.7%)	11 (78.6%)	
None	32 (100%)	39 (100%)		0 (0%)	0 (0%)	
URTI-related medication within 4 week (num. yes, %)	2 (6.3%)	2 (5.1%)	0.839	3 (14.3%)	2 (12.5%)	0.877
